# Daily cannabis use during the novel coronavirus disease (COVID-19) pandemic in Canada: a repeated cross-sectional study from May 2020 to December 2020

**DOI:** 10.1186/s13011-022-00441-x

**Published:** 2022-02-21

**Authors:** Sameer Imtiaz, Samantha Wells, Jürgen Rehm, Christine M. Wickens, Hayley Hamilton, Yeshambel T. Nigatu, Damian Jankowicz, Tara Elton-Marshall

**Affiliations:** 1 Institute for Mental Health Policy Research, Centre for Addiction and Mental Health, 33 Russell Street, ON M5S 2S1 Toronto, Canada; 2 Campbell Family Mental Health Research Institute, Centre for Addiction and Mental Health, 250 College Street, Toronto, ON M5T 1R8 Canada; 3 Dalla Lana School of Public Health, University of Toronto, 6th Floor, 155 College Street, Toronto, ON M5T 3M7 Canada; 4 Department of Epidemiology and Biostatistics, Schulich School of Medicine and Dentistry, Western University, Kresge Building, London, ON N6A 5C1 Canada; 5 Institute of Medical Science, University of Toronto, Room 2374, 1 King’s College Circle, Toronto, ON M5S 1A8 Canada; 6 Department of Psychiatry, University of Toronto, 8th Floor, 250 College Street, Toronto, ON M5T 1R8 Canada; 7 Institute for Clinical Psychology and Psychotherapy, TU Dresden, Chemnitzer Str. 46, 01187 Dresden, Germany; 8 Department of International Health Projects, Institute for Leadership and Health Management, I.M. Sechenov First Moscow State Medical University, Trubetskaya Str., 8, B. 2, 119992 Moscow, Russian Federation; 9 Institute of Health Policy, Management and Evaluation, University of Toronto, 425-155 College Street, Toronto, ON M5T 1P8 Canada; 10 Department of Pharmacology & Toxicology, University of Toronto, 1 King’s College Circle, Toronto, ON M5S 1A8 Canada; 11 Information Management, Centre for Addiction and Mental Health, 1001 Queen Street West, Toronto, ON M6J 1H4 Canada; 12 Department of Health Sciences, Lakehead University, SN 1006, 955 Oliver Road, Thunder Bay, ON P7B 5E1 Canada; 13 School of Epidemiology and Public Health, Faculty of Medicine, University of Ottawa, 600 Peter Morand Crescent, Ottawa, ON K1G 5Z3 Canada

**Keywords:** Canada, SARS-CoV-2, COVID-19, Coronavirus, Cannabis, Marijuana

## Abstract

**Background:**

Daily cannabis use is most strongly implicated in the cannabis-attributable burden of disease. In the context of the novel coronavirus disease (COVID-19) pandemic in Canada, we characterized trends in daily cannabis use in the overall sample and various population subgroups, and examined risk characteristics associated with daily cannabis use.

**Methods:**

A cross-sectional design was operationalized using data from six waves of a national, online survey of adults residing in Canada who spoke English (*N* = 6,021; May-08 2020 to December-01 2020). Trends were characterized using the Cochran-Armitage test and risk characteristics were identified using chi-square test and logistic regression analysis.

**Results:**

Daily cannabis use in the overall sample remained stable (5.34% – 6.10%; *p* = 0.30). This pattern of findings extended to various population subgroups as well. The odds of daily cannabis use were higher for those who: were males (Odds Ratio; 95% Confidence Interval: 1.46; 1.15 – 1.85), were between 18 – 29 years (2.36; 1.56 – 3.57), 30 – 39 years (2.65; 1.93 – 3.64) or 40–49 years (1.74; 1.19 – 2.54), self-identified as white (1.97; 1.47 – 2.64), had less than college or university completion (1.78; 1.39 – 2.28), engaged in heavy episodic drinking (2.05; 1.62 – 2.61), had a job that increased the risk of contracting COVID-19 (1.38; 1.01 – 1.88), experienced loneliness 5–7 days in the past week (1.86; 1.26 – 2.73) and felt very worried (2.08; 1.21 – 3.58) or somewhat worried (1.83; 1.11 – 3.01) about the pandemic’s impact on their financial situation.

**Conclusions:**

Daily cannabis use did not change in the overall sample or various population subgroups during the pandemic. Pandemic-related risks and impacts were associated with daily cannabis use.

**Supplementary Information:**

The online version contains supplementary material available at 10.1186/s13011-022-00441-x.

## Introduction

The novel coronavirus disease (COVID-19) pandemic has resulted in substantial burden of disease in Canada [1]. Government authorities have consequently enacted public health measures to contain the spread of the disease, most prominently physical distancing restrictions that involve closures of non-essential community and business institutions, limitations on in-person socializing and work from home mandates [2]. As these measures are associated with considerable economic and social effects [3], they result in heightened stress, anxiety and loneliness [4]. In addition, they disrupt daily routines by blurring the boundaries between work and leisure. As such, they may ultimately result in changes in cannabis use [5]. Indeed, increases in cannabis use during the pandemic have been documented in Canada [6, 7].

Less is known about near daily or daily cannabis use (referred to here as daily cannabis use), the pattern of consumption most strongly implicated in the cannabis-attributable burden of disease [8, 9]. Indeed, a range of adverse health outcomes have been documented with increasing frequency of cannabis use, including changes in brain structure, neurocognitive effects, mental health problems, cardiovascular problems and motor vehicle injuries [10]. However, trends in daily cannabis use in the overall population and various population subgroups during the pandemic have not been characterized. Based on dollars of sale compiled by Statistics Canada, observed cannabis retail sales compared with projected cannabis retail sales were 25% higher between March 2020 and June 2021, approximating an additional $811 million during the 16-month period [11]. Given the increases in cannabis retail sales, it is important to understand if there are corresponding changes in daily cannabis use as well. According to the National Cannabis Survey, daily cannabis use during the past three months increased among those 15 years and older from 6% in the First Quarter of 2019 to 8% in the Fourth Quarter of 2020 [12, 13]. The Canadian Cannabis Survey on the other hand indicated that daily cannabis use approximated one-fifth of those who used cannabis during the past 12 months in 2020, with no changes observed in this cannabis pattern of consumption from 2019 [14]. More importantly, the roles of the unique circumstances brought on by the pandemic in predicting daily cannabis use are yet to be examined. Such circumstances include employment-related risk of contracting COVID-19, feelings of loneliness, impacts on employment situation and impacts on financial situation, all of which may contribute to daily cannabis use due to a combination of availability of leisure time, feelings of boredom and heightened worries and anxieties. As the current lower-risk cannabis use guidelines recommend cannabis use to not exceed occasional use [10], this knowledge may inform screening and targeted interventions at a time when cannabis is legal and available. We accordingly addressed these critical knowledge gaps using repeated cross-sectional assessments conducted online between May 2020 and December 2020 in Canada. Our specific objectives were as follows:

1. Characterize trends in daily cannabis use in the overall sample and various population subgroups.
2. Examine risk characteristics (including pandemic-related risks and impacts) that are associated with daily cannabis use.

## Materials and methods

### Setting, design and data source

An online, cross-sectional survey of adults (≥ 18 years) residing in Canada was conducted by the Centre for Addiction and Mental Health in collaboration with the market research firm, Delvinia (see Table S1 in Additional File 1 for the Checklist for Reporting Results of Internet E-Surveys [CHERRIES]). The sampling frame was comprised of a million plus members of an existing web panel called AskingCanadians (see http://corporate.askingcanadians.com/ for further details on the web panel). The sampling methodology entailed quota sampling by age, gender and region (proportional to size of the population that spoke English). The survey was repeated at six time points since the pandemic began in 2020: Wave 1 (May 8 – May 12: *N* = 1,005; Completion Rate [CR] = 15.93%), Wave 2 (May 29 – June 01: *N* = 1,002; CR = 17.19%), Wave 3 (June 19 – June 23: *N* = 1,005; CR = 16.40%), Wave 4 (July 10 – July 14: *N* = 1,003; CR = 13.69%), Wave 5 (September 18 – September 22: *N* = 1,003; CR = 17.58%) and Wave 6 (November 27 – December 01: *N* = 1,003; CR = 16.22%) (see Table S2 in Additional File 1 for age, sex and regional compositions of the samples, as well as a comparison of the samples with the general population). Importantly, the sampling methodology ensured that participants were not included more than once in the survey (i.e. one-time inclusion in either of the six survey waves) due to the cross-sectional design. Further details regarding the survey methodology are described in the Supplementary Methods in Additional File 1.

### Measurements

#### Daily cannabis use

Based on responses to the item “During the past seven days, on how many days did you use cannabis (also known as marijuana, hash, pot)?”, daily cannabis use (yes, no) was defined as cannabis use on at least five days during the past week.

#### Demographics

Demographics included gender (male, female), age (18 – 29 years, 30 – 39 years, 40 – 49 years, ≥ 50 years), region (Western [British Columbia, Yukon, Northwest Territories and Nunavut), Prairies [Alberta, Saskatchewan, Manitoba], Central [Ontario] and Atlantic [Quebec, New Brunswick, Newfoundland and Labrador, Nova Scotia, Prince Edward Island]), urbanicity (urban, suburban, rural), marital status (married or living with a partner, widowed, divorced or separated, never married), size of household (1 person, 2 people, 3 people, ≥ 4 people), ethnicity (white, non-white) and education (less than university or college completion, at least university or college completion).

#### Heavy episodic drinking

Heavy episodic drinking (yes, no) was defined as consumption of at least five drinks for men and at least four drinks for women in one drinking occasion during the past week.

#### Pandemic-related risks and impacts

Employment-related risk of contracting COVID-19 was determined by asking participants if they had a job that exposed them to a high risk of contracting COVID-19 (yes, no). Feelings of loneliness were measured by asking participants about the frequency of loneliness during the past week [15]: < 1 day, 1 – 2 days, 3 – 4 days and 5 – 7 days.

The impact of the pandemic on employment situation was determined by asking participants, “How have physical distancing measures due to the COVID-19 pandemic affected your employment situation?”, with the responses coded into six categories: currently working from home, currently not working or loss of employment, previously working from home during the pandemic, previously not working or loss of employment during the pandemic, other and no change. The impact of the pandemic on financial situation was examined by asking participants, “How worried are you about the impact of COVID-19 on your personal financial situation”: not at all worried, not very worried, somewhat worried and very worried.

### Statistical analysis

To maximize the sample size for the statistical analyses, adjacent survey waves were collapsed into periods: Period 1 (Waves 1 – 2), Period 2 (Waves 3 – 4) and Period 3 (Waves 5 – 6). Trends in daily cannabis use in the overall sample were examined using the Cochran-Armitage test. This analysis was then repeated among the various population subgroups to test for differential patterns of change. The examined population subgroups included categories of gender, age, region, urbanicity, marital status, ethnicity, education and heavy episodic drinking. Given the number of simultaneous tests performed, adjustments were made for multiple comparisons using the Bonferroni Correction, a conservative approach that focuses on large and meaningful changes. Thereafter, risk characteristics associated with daily cannabis use were identified using cross-tabulations with chi-square tests. Risk characteristics with *p* < 0.05 in these analyses were subsequently entered in multivariable logistic regression analyses. Variance inflation factors were generated to assess multicollinearity and the Hosmer–Lemeshow test was used to assess the model fit.

## Results

### Trends in daily cannabis use

Daily cannabis use during the pandemic remained stable: 5.34% (*N* = 107) in Period 1, 5.24% (*N* = 105) in Period 2 and 6.10% (*N* = 122) in Period 3 (*p* = 0.2955). After the adjustment for multiple comparisons, differential patterns of change in daily cannabis use were not observed among the various population subgroups (Table 1).

**Table 1:** Trends in Daily Cannabis Use Among Population Subgroups in Canada

	**Period 1 (May 08 to June 1, 2020 [*N* = 107])**^**a**^	**Period 2 (June 19 to July 14, 2020 [*N* = 105])**^**a**^	**Period 3 (September 18 to December 01, 2020 [*N* = 122])**^**a**^	***P*-Value**^**b**^
	**% (N)**	**% (N)**	**% (N)**	
Gender				
Male	32.98 (62)	30.32 (57)	36.70 (69)	0.4878
Female	30.94 (43)	33.81 (47)	35.25 (49)	0.5433
Age				
18—29 Years	36.36 (20)	40.00 (22)	23.64 (13)	0.5223
30—39 Years	27.42 (34)	34.68 (43)	37.90 (47)	0.3080
40—49 Years	34.69 (17)	28.57 (14)	36.73 (18)	0.9181
≥ 50 Years	33.96 (36)	24.53 (26)	41.51 (44)	0.2731
Region				
Western	19.61 (10)	31.37 (16)	49.02 (25)	0.0045
Prairies	34.78 (32)	41.30 (38)	23.91 (22)	0.2309
Central	33.61 (40)	26.05 (31)	40.34 (48)	0.3575
Atlantic	34.72 (25)	27.78 (20)	37.50 (27)	0.8158
Urbanicity				
Urban Area	30.34 (44)	35.17 (51)	34.48 (50)	0.5653
Suburban Area	34.62 (45)	26.15 (34)	39.23 (51)	0.4260
Rural Area	30.51 (18)	33.90 (20)	35.59 (21)	0.6795
Marital Status				
Married or Living with a Partner	29.17 (56)	31.25 (60)	39.58 (76)	0.1384
Widowed, Divorced or Separated	42.50 (17)	25.00 (10)	32.50 (13)	0.6241
Never Married	34.34 (34)	33.33 (33)	32.32 (32)	0.8748
Ethnicity				
White	33.86 (85)	29.08 (73)	37.05 (93)	0.4931
Non-white	28.00 (21)	36.00 (27)	36.00 (27)	0.3878
Education				
Less than College or University Completion	32.06 (42)	32.06 (42)	35.88 (47)	0.4328
At least College or University Completion	32.18 (65)	30.69 (62)	37.13 (75)	0.4184
Heavy Episodic Drinking				
Yes	33.77 (51)	31.79 (48)	34.44 (52)	0.8601
No	30.60 (56)	31.15 (57)	38.25 (70)	0.1597

^**a**^The sum of individual cells may not equal the period totals due to missing data

^**b**^Differences are considered statistically significant at the *p* < 0.0023 level due to the Bonferroni Correction

### Risk characteristics associated with daily cannabis use

Gender, age, marital status, ethnicity, education, heavy episodic drinking, employment-related risk of contracting COVID-19, feelings of loneliness, impacts on employment situation and impacts on financial situation were associated with daily cannabis use in chi-square analyses (Table 2). These risk characteristics were subsequently included in the logistic regression analyses. An adequate model fit was achieved (*p* = 0.4053) and multicollinearity was not detected (Variance Inflation Factors < 10 for all risk characteristics). After the simultaneous adjustment for these risk characteristics, higher odds of daily cannabis use were observed for participants who were males (Odds Ratio [OR]; 95% Confidence Interval [CI]: 1.46; 1.15 – 1.85), between 18 – 29 years (2.36; 1.56 – 3.57), 30 – 39 years (2.65; 1.93 – 3.64) or 40 – 49 years (1.74; 1.19–2.54), self-identified as white (1.97; 1.47 – 2.64), had less than college or university completion (1.78; 1.39 – 2.28) and engaged in heavy episodic drinking (2.05; 1.62 – 2.61). In terms of pandemic-related risks and impacts, increased odds of daily cannabis use were demonstrated for participants who had a job that increased the risk of contracting COVID-19 (OR, 95% CI: 1.38; 1.01 – 1.88), experienced loneliness 5 to 7 days during the past week (1.86; 1.26 – 2.73), and felt very worried (2.08; 1.21 – 3.58) or somewhat worried (1.83; 1.11 – 3.01) about the impact of the pandemic on financial situation.

**Table 2:** Risk Characteristics Associated with Daily Cannabis Use in Canada

	**Daily Cannabis Use**			**Unadjusted Odds Ratio**^**a**^		**Adjusted Odds Ratio**^**a**^**,**^**b**^	
	**N**	**%**	***P*-Value**	**Estimate**	**95% CI**	**Estimate**	**95% CI**
Gender							
Male	188	6.32	**0.0115**	**1.38**	**1.10 – 1.73**	**1.46**	**1.15—1.85**
Female	146	4.82		Reference		Reference	
Age							
18—29 years	55	7.47	** < 0.0001**	**2.07**	**1.48 – 2.89**	**2.36**	**1.56—3.57**
30—39 years	124	7.72		**2.14**	**1.64 – 2.80**	**2.65**	**1.93—3.64**
40—49 years	49	5.80		**1.58**	**1.11 – 2.23**	**1.74**	**1.19—2.54**
≥ 50 years	106	3.76		Reference		Reference	
Region							
Western	51	5.62	0.1080				
Prairies	92	6.30					
Central	119	4.75					
Atlantic	72	6.37					
Urbanicity							
Urban area	145	5.17	0.4387				
Suburban area	130	5.82					
Rural area	59	6.10					
Marital Status							
Married or living with a partner	192	5.11	**0.0456**	**0.73**	**0.57 – 0.94**	0.96	0.71—1.29
Widowed, divorced or separated	40	5.48		0.79	0.54 – 1.15	1.14	0.74—1.77
Never married	99	6.88		Reference		Reference	
Size of household							
1 person	66	5.37	0.6592				
2 people	134	5.34					
3 people	67	6.36					
≥ 4 people	67	5.61					
Ethnicity							
White	251	6.02	**0.0199**	**1.37**	**1.05 – 1.78**	**1.97**	**1.47 – 2.64**
Non-white	75	4.47		Reference		Reference	
Education							
Less than college or university completion	131	8.13	** < 0.0001**	**1.81**	**1.44 – 2.28**	**1.78**	**1.39—2.28**
At least college or university completion	202	4.65		Reference		Reference	
Heavy Episodic Drinking							
Yes	151	9.84	** < 0.0001**	**2.42**	**1.93 – 3.04**	**2.05**	**1.62—2.61**
No	183	4.11		Reference		Reference	
Employment-Related Health Risk							
Yes	60	8.04	**0.0016**	**1.59**	**1.19 – 2.13**	**1.38**	**1.01 – 1.88**
No	274	5.21		Reference		Reference	
Feelings of Loneliness							
5—7 Days	59	11.59	** < 0.0001**	**2.77**	**2.01 – 3.82**	**1.86**	**1.26—2.73**
3—4 Days	61	7.31		**1.67**	**1.22 – 2.27**	1.28	0.91—1.79
1—2 Days	74	4.73		1.05	0.79 – 1.40	0.91	0.67—1.23
< 1 Day	140	4.52		Reference		Reference	
Impact of Pandemic on Financial Situation							
Very worried	88	7.46	**0.0020**	**2.28**	**1.40 – 3.71**	**2.08**	**1.21—3.58**
Somewhat worried	146	5.60		**1.68**	**1.05 – 2.67**	**1.83**	**1.11 – 3.01**
Not very worried	79	4.93		1.47	0.90 – 2.34	1.68	1.00—2.82
Not at all worried	21	3.41		Reference		Reference	
Impact of Pandemic on Employment Situation							
Currently working from home	71	4.77	**0.0233**	0.92	0.69 – 1.23	0.81	0.59—1.12
Currently not working or loss of employment	49	7.35		**1.46**	**1.05 – 2.03**	1.01	0.70—1.46
Previously working from home	13	5.06		0.98	0.55 – 1.75	0.77	0.42—1.40
Previously not working or loss of employment	19	7.79		1.55	0.95 – 2.55	0.81	0.46—1.43
Other	26	8.05		**1.61**	**1.05 – 2.48**	1.52	0.96—2.42
No Impact	156	5.16		Reference		Reference	

Note: Bolded interface denotes *p* < 0.05

*Abbreviations*: *CI* Confidence Interval

^**a**^The outcome reference category in all logistic regression models is no daily cannabis use

^**b**^The odds ratios are adjusted for gender, age, marital status, ethnicity, education, heavy episodic drinking, employment-related health risk, feelings of loneliness, impact of the pandemic on employment situation and impact of pandemic on financial situation

## Discussion

We characterized trends in daily cannabis use and examined risk characteristics associated with daily cannabis use during the pandemic in Canada. Daily cannabis use in the overall sample and various population subgroups did not change. Risk characteristics associated with daily cannabis use included gender, age, ethnicity, education, heavy episodic drinking, employment-related risk of contracting COVID-19, feelings of loneliness and impacts on financial situation.

Trends in daily cannabis use during the pandemic have not been previously characterized in Canada, limiting direct comparisons with the present findings. The National Cannabis Survey most recently demonstrated that daily cannabis use during the past three months approximated 8% among those 15 years and older in the Fourth Quarter of 2020 [13]. Despite the differences in the assessment time frame (past three months vs. past week), these findings are broadly similar to the estimate obtained hereunder for Period 3 (6%). On the other hand, changes in cannabis use since the onset of the pandemic have been most frequently examined, with increases in cannabis use documented in the overall population [16, 17] and among those who use cannabis [7, 18, 19]. Based on a representative sample of adults (≥ 25 years), cannabis use during the pandemic increased in 5%, decreased in 2% and remained stable in 93% of the population between March 29, 2020 and April 03, 2020 in Canada [16]. An earlier analysis of the first three waves of the present survey also demonstrated that cannabis use increased in 52% of those who use cannabis compared to before the beginning of the pandemic between May 08, 2020 and June 23, 2020 in Canada [7]. These findings collectively indicate increased cannabis use during the pandemic. Coupled with the present null findings pertaining to the trends in daily cannabis use, it is possible that the frequency of cannabis use increased, but not to an extent of daily cannabis use. Indeed, median number of days of cannabis use in the present survey were 4 days in Period 1, 3 days in Period 2 and 4 days in Period 3. Alternatively, changes in daily cannabis use may have occurred that were not captured by the surveys, as the first survey wave was conducted well after the enactment of the initial public health measures in March 2020, or changes may have occurred in other cannabis patterns of consumption.

In terms of risk characteristics associated with daily cannabis use during the pandemic, the observed effects of male gender [20], younger age [20] and lesser education [21] are consistent with assessments of cannabis patterns of consumption that were conducted before the pandemic. The same is also applicable to heavy episodic drinking. In the context of co-occurring alcohol and cannabis use, higher levels of consumption of one substance are related to higher levels of consumption of the other substance [22]. Importantly, co-occurring cannabis and alcohol use is associated with greater harms and consequences than either substance alone [22]. Pandemic-related risks and impacts in relation to daily cannabis use have been examined to a lesser extent. As many have experienced employment-related risk of contracting COVID-19, feelings of loneliness and impacts on financial situation during the pandemic, some may engage in substance use to avoid and cope with the resulting negative affect, including stress, depression and anxiety. Indeed, self-isolation and coping with depression motives were both associated with cannabis use levels during the pandemic when accounting for cannabis use levels before the pandemic [23]. Compared to their counterparts who did not engage in self-isolation, those who did engage in self-isolation were using 20% more cannabis [23]. Impacts on financial situation have contrastingly yielded mixed findings. Reporting “too soon to determine financial impacts” and “experiencing financial impacts” were associated with both an increase and decrease in cannabis use among the general population [16]. However, in an earlier analysis of the first three waves of the present survey, being “somewhat worried” about the impacts on financial situation was associated with an increase in cannabis use among those who use cannabis [7]. Although these findings are not directly comparable due to a different outcome, they are consistent with the positive association observed between impacts on financial situation and daily cannabis use.

Further research is nonetheless needed to obtain a broader understanding of the changes in cannabis patterns of consumption due to COVID-19. Daily cannabis use needs to be monitored among certain high-risk segments of the population. Indeed, increase in cannabis use and problematic cannabis use were elevated among those with mental health concerns and substance use concerns (including histories of psychiatric disorders) in Canada [18]. In addition, the intersection of coping motives with pandemic-related risks and impacts in predicting daily cannabis use warrant further exploration. Furthermore, the trajectory of cannabis patterns of consumption other than daily cannabis use need to be examined, such as frequency per day, quantity per occasion, modes of administration and types of products, all of which may impact the resulting cannabis-attributable burden of disease. Unfortunately, such assessments were not included in the present surveys, but they represent important lines of investigation for future studies.

There are some limitations that should be considered. First, given the absence of a measurement before the pandemic began, it is not possible to determine if daily cannabis use changed due to the pandemic. Furthermore, the recent legalization of recreational cannabis consumption in October 2018 in Canada limits the comparison with historic data, and serves as a confounder because the expanding cannabis retail market may have also affected the trends in daily cannabis use. Second, although the effects are expected to be minimal since the surveys were conducted online rather than in-person or over the telephone, the self-reported nature of the data may have resulted in social desirability and recall biases. As such, daily cannabis use may have been underreported. However, self-reports of alcohol and drug use have been shown to be valid [24]. Third, causal inferences between the risk characteristics and daily cannabis use cannot be made due to a cross-sectional design that does not account for temporality. Fourth, certain geographic segments may not have been adequately represented, as the survey was restricted to the population that spoke English. Fifth, owing to a sampling frame comprised of an existing web panel, sampling strategy lacking random selection procedures, recruitment of a modest number of participants and an average survey completion rate of 16%, the generalizability of the findings may be limited. These concerns about limited generalizability may be especially pertinent to those without internet access. However, the effects are expected to be minimal, as quota sampling in online surveys is an established method to rapidly collect data concerning sensitive subjects [25–27], and only about 6% of the population reports a lack of home internet access [28]. Sixth, a distinction between non-medicinal and medicinal cannabis use was not made, which would affect rates of daily cannabis use. Finally, feelings of loneliness captured by the surveys may or may not have been precipitated due to the pandemic, as it was not specified in the assessment measure.

## Conclusions

Daily cannabis use in the overall sample and various population subgroups remained stable during the pandemic in Canada. Pandemic-related risks and impacts were associated with daily cannabis use. As increased frequency of cannabis use is linked to acute and chronic adverse health outcomes [29, 30] and the current lower-risk guidelines recommend no more than occasional use for those who use cannabis [10], it is imperative for government authorities to ensure non-medicinal daily cannabis use remains limited, especially as multiple lines of inquiries suggest an increase in cannabis use. These findings can inform screening and targeted interventions to reduce daily cannabis use in Canada.

## Supplementary Information

**Additional file 1.**

## Data Availability

Survey data are publicly available from the Methodify Platform by Delvinia (https://www.delvinia.com/camh-coronavirus-mental-health/).

## Abbreviations

CHERRIES: Checklist for the Checklist for Reporting Results of Internet E-Surveys
COVID-19: Novel Coronavirus Disease
CR: Completion Rate
OR: Odds Ratio
