# Supplementary material for: Daily cannabis use during the novel coronavirus disease (COVID-19) pandemic in Canada: a repeated cross-sectional study from May 2020 to December 2020
Source: Subst Abuse Treat Prev Policy. 2022 Feb 21;17:14. doi: 10.1186/s13011-022-00441-x (PMC8860264; doi:10.1186/s13011-022-00441-x)
Supplement: Supplementary file 1 — Additional file 1. [file 13011_2022_441_MOESM1_ESM.docx]

**Supplementary Appendix To:** Daily Cannabis Use During the Novel Coronavirus Disease (COVID-19) Pandemic in Canada: A Repeated Cross-Sectional Study From May 2020 to December 2020

**Supplementary Methods**

**Survey Design**

The surveys sampled members of an existing web panel called AskingCanadians through quota sampling by age, gender and region (proportional to the population that spoke English), who were recruited from loyalty rewards programs with major corporations (e.g. Walmart, Hudson Bay, Petro Canada). These surveys aimed to represent the population that spoke English in Canada. Although these surveys included convenience samples, they were considered closed surveys, as they were not available to the general public. Survey weights were not generated or applied. For their voluntary participation in the surveys, members of the web panel were compensated with their desired loyalty reward program points.

**Survey Development and Testing**

The surveys were developed and conducted in collaboration between the Centre for Addiction and Mental Health and Delvinia. Surveys were built off an in-house survey template from Delvinia, which was tested for technical functionality and designed to be user friendly across multiple platforms (e.g. desktops, mobile devices, tablet devices). Importantly, technical errors were detected through internal systems (cofirmit error reporting), and participants were able to initiate contact through customer support email.

**Survey Procedures**

Based on age, gender and region compositions, selected members of the web panel were sent an email inviting them to complete the surveys. The email highlighted the reason for the survey, approximate time required to fill the survey, number of points provided for completing the survey and the closing date of the survey. After clicking on an embedded, single-entry, unique weblink for the survey in the email, an informed consent form was presented that provided additional details, including study purpose, study methods, participant data confidentiality, participant legal rights and principal investigator contact information. After review of the informed consent form, an opportunity was provided to opt-out of the survey. Members of the web panel then proceeded to the survey, where they entered responses to the survey items into the online platform. Personal information of the members of the web panel was housed on a separate server, with restricted access given to limited employees of Delvinia. No additional personal information was collected or stored for the surveys.

**Survey Administration**

Survey items were not randomized or alternated, included short response lists and utilized adaptive questioning (where certain survey items were conditionally displayed based on responses to other survey items). One survey item was displayed per screen, with one screen comprising the informed consent form and a total of 30 screens comprising the survey. As participants progressed through the survey, they were not allowed to return back to previous screens and change the responses, as the back button was disabled. Participants were made aware of this setup upon entry to the surveys. Response fields on all screens incorporated forced entries, meaning respondents could not proceed further until they responded to the item on the screen. A cut-off time point for completing the surveys was not applied. Surveys were monitored by a project manager throughout the fielding process, checked for completeness at multiple time points and underwent a final check to ensure all records were complete. Surveys that included responses to all items were considered complete and included in the final database. Once the participants completed the surveys, they were not able to access the surveys again through their user accounts. Given the closed design of the survey, internet cookies were not used to assign unique identifiers, neither were computer IP checks implemented, towards the mitigation of multiple entries from the same participants. Importantly, the web panel is proactively maintained to be free of duplicate user accounts.

**Survey Completion Rate and Survey Participation Rate**

Survey completion rates were calculated as the number of complete surveys divided by the estimated number of eligible participants. Survey participation rates were calculated as the number of complete and incomplete surveys divided by the estimated number of eligible participants. The estimated number of eligible participants were calculated as follows: (number of survey invites sent) – (number of participants excluded due to filled quota) – (number of participants excluded after screening). The survey completion rates and survey participation rates were 16.06% and 18.00%, respectively for Waves 1 to 6, 15.93% and 17.66%, respectively for Wave 1, 17.19% and 19.41%, respectively for Wave 2, 16.40% and 18.26%, respectively for Wave 3, 13.69% and 15.22% respectively for Wave 4, 17.58% and 20.00%, respectively for Wave 5, 16.22% and 18.22%, respectively for Wave 6.

**Supplementary Tables**

**Table S1: Checklist for Reporting Results of Internet E-Surveys (CHERRIES)**

|  |  |  |
| --- | --- | --- |
| **Item Category** | **Checklist Item** | **Reported In** |
|  |  |  |
| **Design** | Describe survey design | Methods – Setting, Design and Data Source; Supplementary Methods – Survey Design |
|  |  |  |
| **IRB (Institutional Review Board) Approval and Informed Consent Process** | IRB approval | Methods - Ethics Approval |
|  |  |  |
|  | Informed consent | Supplementary Methods – Survey Procedures |
|  |  |  |
|  | Data protection | Supplementary Methods – Survey Procedures |
|  |  |  |
| **Development and Pre-Testing** | Development and testing | Supplementary Methods – Survey Development and Testing |
|  |  |  |
| **Recruitment Process and Description of the Sample Having Access to the Questionnaire** | Open survey versus closed survey | Supplementary Methods – Survey Design |
|  |  |  |
|  | Contact mode | Supplementary Methods – Survey Design and Survey Procedures |
|  |  |  |
|  | Advertising the survey | Supplementary Methods – Survey Design and Survey Procedures |
|  |  |  |
| **Survey Administration** | Web/E-mail | Supplementary Methods – Survey Procedures |
|  |  |  |
|  | Context | Supplementary Methods – Survey Design and Survey Procedures |
|  |  |  |
|  | Mandatory/voluntary | Supplementary Methods – Survey Design |
|  |  |  |
|  | Incentives | Supplementary Methods – Survey Design |
|  |  |  |
|  | Time/date | Methods – Setting, Design and Data Source |
|  |  |  |
|  | Randomization of items or questionnaires | Supplementary Methods – Survey Administration |
|  |  |  |
|  | Adaptive questioning | Supplementary Methods – Survey Administration |
|  |  |  |
|  | Number of items | Supplementary Methods – Survey Administration |
|  |  |  |
|  | Number of screens | Supplementary Methods – Survey Administration |
|  |  |  |
|  | Comprehensiveness check | Supplementary Methods – Survey Administration |
|  |  |  |
|  | Review step | Supplementary Methods – Survey Administration |
|  |  |  |
| **Response Rates** | Unique site visitor | Supplementary Methods – Survey Design, Survey Procedures and Survey Administration |
|  |  |  |
|  | View rate (ratio of unique survey visitors/unique site visitors) | Not computed (See Supplementary Methods – Survey Completion Rate and Survey Participation Rate) |
|  |  |  |
|  | Participation rate (ratio of unique visitors who agreed to participate/unique first survey page visitors) | Not computed (See Supplementary Methods – Survey Completion Rate and Survey Participation Rate) |
|  |  |  |
|  | Completion rate (ratio of users who finished the survey/users who agreed to participate) | Not computed (See Supplementary Methods – Survey Completion Rate and Survey Participation Rate) |
|  |  |  |
| **Preventing Multiple Entries from the Same Individual** | Cookies used | Supplementary Methods – Survey Administration |
|  |  |  |
|  | IP check | Supplementary Methods – Survey Administration |
|  |  |  |
|  | Log file analysis | Supplementary Methods – Survey Administration |
|  |  |  |
|  | Registration | Supplementary Methods – Survey Procedures and Survey Administration |
|  |  |  |
| **Analysis** | Handling of incomplete questionnaires | Supplementary Methods – Survey Administration |
|  |  |  |
|  | Questionnaires submitted with an atypical timestamp | Supplementary Methods – Survey Administration |
|  |  |  |
|  | Statistical correction | Supplementary Methods – Survey Design |
|  |  |  |

**Table S2: Comparisons of Age, Sex and Region in the Surveys with Population Estimates from on July 1^st^, 2020 from Statistics Canada**

|  |  | |  | |  | |  | |  | |  | |  | |
| --- | --- | --- | --- | --- | --- | --- | --- | --- | --- | --- | --- | --- | --- | --- |
|  | **Statistics Canada Population Estimates on July 01, 2020^^^** | | **Wave 1** | | **Wave 2** | | **Wave 3** | | **Wave 4** | | **Wave 5** | | **Wave 6** | |
|  | **N** | **%** | **N** | **%** | **N** | **%** | **N** | **%** | **N** | **%** | **N** | **%** | **N** | **%** |
|  |  |  |  |  |  |  |  |  |  |  |  |  |  |  |
|  |  |  |  |  |  |  |  |  |  |  |  |  |  |  |
|  |  |  |  |  |  |  |  |  |  |  |  |  |  |  |
| **Age** |  |  |  |  |  |  |  |  |  |  |  |  |  |  |
| 18 to 29 | 6,017,203 | 19.6 | 132 | 13.1 | 130 | 13.0 | 141 | 14.0 | 125 | 12.5 | 127 | 12.7 | 82 | 8.2 |
| 30 to 39 | 5,292,403 | 17.2 | 262 | 26.1 | 259 | 25.9 | 253 | 25.2 | 263 | 26.2 | 263 | 26.2 | 310 | 30.9 |
| 40 to 49 | 4,854,363 | 15.8 | 129 | 12.8 | 136 | 13.6 | 139 | 13.8 | 152 | 15.2 | 152 | 15.2 | 143 | 14.3 |
| 50 to 59 | 5,194,811 | 16.9 | 177 | 17.6 | 176 | 17.6 | 168 | 16.7 | 157 | 15.7 | 153 | 15.3 | 162 | 16.2 |
| 60 to 69 | 4,727,516 | 15.4 | 192 | 19.1 | 178 | 17.8 | 174 | 17.3 | 189 | 18.8 | 182 | 18.2 | 192 | 19.1 |
| 70+ | 4,668,591 | 15.2 | 113 | 11.2 | 123 | 12.3 | 130 | 12.9 | 117 | 11.7 | 126 | 12.6 | 114 | 11.4 |
| Total | 30,754,887 | 100.0 | 1005 | 100.0 | 1002 | 100.0 | 1005 | 100.0 | 1003 | 100.0 | 1003 | 100.0 | 1003 | 100.0 |
|  |  |  |  |  |  |  |  |  |  |  |  |  |  |  |
| **Sex^#^** |  |  |  |  |  |  |  |  |  |  |  |  |  |  |
| Male | 15,181,112 | 49.4 | 504 | 50.3 | 492 | 49.8 | 501 | 50.1 | 501 | 50.5 | 497 | 50.0 | 492 | 49.5 |
| Female | 15,573,775 | 50.6 | 498 | 49.7 | 497 | 50.3 | 499 | 49.9 | 492 | 49.6 | 498 | 50.1 | 503 | 50.6 |
| Total | 30,754,887 | 100 | 1002 | 100.0 | 989 | 100.0 | 1000 | 100.0 | 993 | 100.0 | 995 | 100.0 | 995 | 100.0 |
|  |  |  |  |  |  |  |  |  |  |  |  |  |  |  |
| **Region** |  |  |  |  |  |  |  |  |  |  |  |  |  |  |
| Alberta | 3,445,146 | 11.2 | 140 | 13.9 | 140 | 14.0 | 140 | 13.9 | 133 | 13.3 | 137 | 13.7 | 141 | 14.1 |
| British Columbia | 4,273,972 | 13.9 | 152 | 15.1 | 146 | 14.6 | 150 | 14.9 | 151 | 15.1 | 148 | 14.8 | 152 | 15.2 |
| Manitoba | 1,068,553 | 3.5 | 67 | 6.7 | 62 | 6.2 | 49 | 4.9 | 51 | 5.1 | 70 | 7.0 | 65 | 6.5 |
| New Brunswick | 645,289 | 2.1 | 22 | 2.2 | 34 | 3.4 | 33 | 3.3 | 34 | 3.4 | 34 | 3.4 | 33 | 3.3 |
| Newfoundland and Labrador | 436,312 | 1.4 | 28 | 2.8 | 27 | 2.7 | 18 | 1.8 | 23 | 2.3 | 18 | 1.8 | 19 | 1.9 |
| Northwest Territories | 34,430 | 0.1 | 2 | 0.2 | 0 | 0.0 | 2 | 0.2 | 0 | 0.0 | 1 | 0.1 | 1 | 0.1 |
| Nova Scotia | 813,389 | 2.6 | 52 | 5.2 | 53 | 5.3 | 51 | 5.1 | 56 | 5.6 | 65 | 6.5 | 40 | 4.0 |
| Nunavut |  |  |  |  |  |  |  |  | 1 | 0.1 | 1 | 0.1 | 0 | 0.0 |
| Ontario | 11,971,129 | 38.9 | 418 | 41.6 | 418 | 41.7 | 415 | 41.3 | 421 | 42 | 419 | 41.8 | 419 | 41.8 |
| Prince Edward Island | 129,799 | 0.4 | 23 | 2.3 | 21 | 2.1 | 18 | 1.8 | 11 | 1.1 | 11 | 1.1 | 19 | 1.9 |
| Quebec | 6,972,707 | 22.7 | 57 | 5.7 | 55 | 5.5 | 72 | 7.2 | 68 | 6.8 | 63 | 6.3 | 76 | 7.6 |
| Saskatchewan | 905,623 | 2.9 | 44 | 4.4 | 46 | 4.6 | 55 | 5.5 | 54 | 5.4 | 36 | 3.6 | 37 | 3.7 |
| Yukon | 33,660 | 0.1 | 0 | 0.0 | 0 | 0.0 | 2 | 0.2 | 0 | 0.0 | 0 | 0.0 | 1 | 0.1 |
| Total | 30,754,887 | 100.0 | 1005 | 100.0 | 1002 | 100.0 | 1005 | 100.0 | 1003 | 100.0 | 1003 | 100.0 | 1003 | 100.0 |
|  |  |  |  |  |  |  |  |  |  |  |  |  |  |  |

**^^^** Statistics Canada population estimates on July 01, 2020 were obtained from Statistics Canada Table: 17-10-0005-01 (https://www150.statcan.gc.ca/t1/tbl1/en/tv.action?pid=1710000501)

**^#^** Other gender identities from the surveys were excluded in the cross-tabulations as estimates for them are not available from Statistics Canada
